# Synergistic Protective Effect of Konjac Mannan Oligosaccharides and *Bacillus subtilis* on Intestinal Epithelial Barrier Dysfunction in Caco-2 Cell Model and Mice Model of Lipopolysaccharide Stimulation

**DOI:** 10.3389/fimmu.2021.696148

**Published:** 2021-09-17

**Authors:** Lupeng Chen, Shuai Zhang, Shi Wu, Zhuqing Ren, Guoquan Liu, Jian Wu

**Affiliations:** College of Animal Sciences & Technology/College of Veterinary Medicine, Huazhong Agricultural University, Wuhan, China

**Keywords:** *Bacillus subtilis*, KMOS, Caco-2 cells, LPS, intestinal injury

## Abstract

As the first line of defense against intestinal bacteria and toxins, intestinal epithelial cells are always exposed to bacteria or lipopolysaccharide (LPS), whereas pathogenic bacteria or LPS can cause intestinal epithelial cell damage. Previous studies have shown that konjac mannan oligosaccharides (KMOS) have a positive effect on maintaining intestinal integrity, and *Bacillus subtilis* (BS) can promote the barrier effect of the intestine. However, it is still unknown whether KMOS and BS have a synergistic protective effect on the intestines. In this study, we used the LPS-induced Caco-2 cell injury model and mouse intestinal injury model to study the synergistic effects of KMOS and BS. Compared with KMOS or BS alone, co-treatment with KMOS and BS significantly enhanced the activity and antioxidant capacity of Caco-2 cell, protected mouse liver and ileum from LPS-induced oxidative damage, and repaired tight junction and mucus barrier damage by up-regulating the expression of Claudin-1, ZO-1 and MUC-2. Our results demonstrate that the combination of KMOS and BS has a synergistic repair effect on inflammatory and oxidative damage of Caco-2 cells and aIIeviates LPS-induced acute intestinal injury in mice.

## Introduction

The intestinal tract, as the largest bacterial endotoxin reservoir in the body, has a complete intestinal epithelial barrier, which plays an important role in maintaining the permeability of epithelial cells and homeostasis of the internal environment of the body (1). Intestinal epithelial cells are an important part of the gut mucosal barrier, the first line of defense against intestinal toxins and bacteria (2, 3), and play an important regulatory role in the immune system of the host (4). Tight junctions (TJs) are the primary factors that determine paracellular permeability (5). The barrier and permeability properties of TJs are defined in a significant part by the ensemble of claudin proteins expressed (6). The cytoplasmic protein, zonula occludens-1 (ZO-1), is a key protein that maintains TJ structure and intestinal epithelial barrier function (7). In the colonic mucosa, the main mucin gene is *MUC2*, a secretory mucin, and to a lesser extent MUC1, MUC3, and MUC4, which are both transmembrane mucins and secretory mucins from the splicing variants (8).

Oxidative stress is closely associated with inflammation and immunity. Intestinal epithelial cells are strongly involved in mucosal oxidative stress and inflammatory responses (9). Nuclear factor E2-related factor 2 (Nrf2) is a central regulator of cells against oxidative stress. High levels of lipopolysaccharide (LPS) are present in the colon. LPS not only causes inflammation damage, but also induces oxidative stress (10), causing damage to the intestinal structure and function (11). LPS is often used to induce intestinal epithelial barrier dysfunction in a Caco-2 cell model (12) and a pathological mouse model (13).

Konjac mannan oligosaccharides (KMOS) are important functional oligosaccharides, which are the hydrolytic products of konjac glucomannan, isolated from tubers of *Amorphophallus konjac* K. Koch (14). Konjac mannan oligosaccharide (KMOS) is a hydrolysis product of konjac glucomannan, consisting of β-D-mannose and β-D-glucose residues linked together by β-(1→4) glycosidic bonds. Recently, the protective effects of KMOS on intestinal immunity and integrity have received much attention. KMOS supplementation has been reported to help improve trinitrobenzenesulfonic acid-induced colitis and improve the intestinal condition of patients with inflammatory bowel disease (IBD) (15). Furthermore, KMOS supplementation improved colonic epithelial integrity and blocked the production of pro-inflammatory cytokines including IL-1β, IL-6 and TNF-α, indicating the inhibition of intestinal inflammation by KMOS (16). Recent studies suggest that KMOS amelioration of DSS-induced colitis requires activation of the SIGNR1 signaling pathway, and that activation of this signal is critical for KMOS-mediated macrophage phenotype switching (16). KMOS deliver various physiological functions, such as dietary fiber and prebiotics (17), regulation of immune system (18), antiobesity (19), and attenuation of glucose metabolism dysfunction (18). In particular, Liu et al. (15) indicated that konjac oligosaccharide is an anti-inflammatory agent and could be useful as a prebiotic to design functional foods for ulcerative colitis. Jian et al. (20) found a protective effect of Konjac oligo-glucomannan against H_2_O_2_-induced oxidative damage in a human hepatic cell line. The inclusion of Konjac flour in the gestation diet changes the gut microbiota, alleviates oxidative stress, and improves insulin sensitivity in sows (21). However, the exact molecular mechanisms involved in the anti-inflammatory and antioxidant effects of KMOS in intestinal epithelial cells remain poorly understood.

Probiotics can regulate intestinal flora and protect against intestinal injury. *Bacillus subtilis* is a biosafety bacterium used in many studies (22) and has extensive applications. In particular, there have been several studies on the protective effect of BS on intestinal barrier function. Musa et al. (23) indicated that *Bacillus subtilis* B21 improves the intestinal health and performance of broiler chickens with *Clostridium perfringens*-induced necrotic enteritis. Supplementation of the spores of BS and *Bacillus coagulans* improved growth performance and was beneficial to the intestinal microbiota in rats (24). It was shown that oral administration of Bacillus subtilis fermented milk could reduce intestinal mucosal injury and inflammatory response and induce intestinal stem cell proliferation to promote the reconstruction of mucosal barrier. In addition, Bacillus subtilis can rebalance the intestinal flora, such as increasing the abundance of Bacillus, Alistipes and Lactobacillus, while decreasing the abundance of Escherichia coli (25) and Bacillus mimicus (26), as well as the relative number of E. coli. Bacillus subtilis increased ZO-1 protein expression, attenuated tight junction damage, and reduced apoptosis. Mechanistically, BS may have protected ZO-1 protein by activating toll-like receptor signaling pathways and reduced damage by downregulating death receptor genes and upregulating DNA repair genes (27).

However, there are still many bottleneck problems in the research and application of probiotics, especially the rapid colonization, growth, and maintenance of a rich concentration of bacteria in the body after addition. In practice, the application of probiotics alone cannot achieve a good and stable effect. However, whether KMOS and BS have synergistic protective effects on the intestinal tract, and the mechanism of their effects, are still unknown. In the present study, we detected the synergistic protective effect of KMOS and BS against LPS-induced intestinal epithelial cell injury in a Caco-2 cell model and a mouse model, respectively. Our study shows that the combined use of KMOS and BS has a synergistic repair effect on Caco-2 cells and intestinal injury in mice, can enhance the antioxidant function of cells, and has a good protective effect against LPS-induced acute injury in mice.

## Materials and Methods

### Cell Culture and Treatment

When the Caco-2 cells covered the monolayer, the old medium was discarded and the cells were rinsed with sterile PBS three times. Next, 2 mL of 0.125% trypsin was added to digest the cells, the rounding of the cells was observed under the microscope, and discarded when the cell gap increased. The trypsin was then removed and a certain amount of high-sugar DMEM basal medium containing 10% FBS was added to terminate the digestion. The suspension was repeatedly pipetted until the cells were completely shed and dispersed into single cells, cultured in separate bottles, and placed in a 5% CO_2_ incubator at 37°C. The fluid was changed every 2–3 days. After 70%–90% of the cells adhered to the wall, they were seeded on a 6-well plate and the cells were processed after 48 h.

In this study, Caco-2 cells were treated with different concentrations of LPS, and the optimal concentration and duration of LPS treatment were determined by detecting the expression of relevant inflammatory factors and changes of oxidation indexes at different time periods, so as to construct the inflammatory injury model and oxidative damage model of Caco-2 cells, respectively. In the LPS group, Caco-2 cells was diluted in ddH_2_O with LPS (Sigma, USA). In the negative control experiments, ddH_2_O was added to untreated cells as a vehicle group. In the model experiment of inflammatory injury of Caco-2 cells, LPS with concentration of 1 μg/mL, KMOS and BS were added simultaneously for 6h. In the model experiment of oxidative injury, Caco-2 cells were treated with 2 μg/mL LPS, KMOS and BS at the same time for 8h. In all cell experiments, the supplemental concentrations of KMOS and BS were 2 g/L and 10^7^ CFU/mL, respectively. The culture conditions of *Bacillus subtilis* in the LPS+BS treatment group were normal LB liquid medium, while the LPS+KMOS+BS treatment group were LB liquid medium containing 2 g/L konjac mannan oligosaccharide. All the bacterial broths were incubated in a shaker at 200 rpm 37°C for 24h. The cells used in this study were human colon cancer epithelial cells (Caco-2 cells) from the Huazhong Agricultural University College of Fisheries. The KMOS used in the experiment was extracted from konjac refined powder and purchased from Enshi Tiantianjia Biotechnology Co., Ltd. BS was isolated from pig manure and has been deposited in China Center for Type Culture Collection (CCTCC) and the CCTCC NO: M2019185.

### MTT Method to Detect Cell Viability

MTT colorimetry is an effective method for detecting cell survival and growth. The detection principle is that exogenous MTT can be reduced by succinate dehydrogenase in the mitochondria of living cells to water-insoluble blue-purple crystal formazan and deposited in the cells, but dead cells have no such function. Dimethyl sulfoxide (DMSO) can dissolve formazan in cells, and its absorbance was measured by enzyme-linked immunosorbent assay (ELISA) at 490 nm, which can indirectly reflect the number of living cells. In a certain range of cell numbers, the amount of MTT crystallization is proportional to the number of cells. The cell suspension was prepared and placed into a 96-well cell culture plate, 100 µL was added to each well (the control well and zero adjustment hole was set at the same time), 100 µL of the concentration gradient drug was added after the cells were covered at the bottom of the well, and then incubated for 24 h. Next, the cells were rinsed two times with PBS, 100 µL of 0.5% MTT medium was added to each well, and incubated for 4 h in the dark. After, 150 µL DMSO was added to each well, shaken at low speed for 10 min on a shaker, and left until the crystals are fully dissolved. The absorbance of each hole was measured at 490 nm wavelength.

### Western Blotting

Tissues or cells were collected and homogenized in a lysis buffer with a handheld homogenizer to prepare a lysis solution. The total protein of the cells and tissues was extracted, and the protein concentration was determined using the Bradford method. The homologs were separated using sodium dodecyl sulfate-polyacrylamide gel electrophoresis (SDS-PAGE) and then transferred to polyvinylidene fluoride membranes. The membranes and antibodies were incubated overnight at 4°C. The samples were then washed twice with TBST (TBS containing 0.05% Tween - 20) and once with TBS (Tris-HCl Buffered Saline) for 10 min each time. The secondary antibody was incubated at room 37°C for 1 h, and chemiluminescence imaging was performed. The following antibodies were used: anti-claudin-1, anti-ZO-1 (Cell Signaling Technologies), anti-SOD, anti-Nrf2 (Proteintech, Wuhan, China), horseradish peroxidase (HRP)-labeled goat anti-rabbit IgG (Proteintech, Wuhan, China), and HRP-labeled goat anti-mouse IgG (Proteintech, Wuhan, China).

### Real-Time PCR

We used the QuantStudio6Flex real-time polymerase chain reaction system (ABI, Thermo Fisher, Shanghai, China) and Roche LightCycler^®^480 (Roche, Switzerland), and the following PCR program for real-time polymerase chain reaction: denaturation at 95°C for 10 min, expansion at 95°C for 45 cycles for 15s, and annealing and extension at 60°C for 1 min. Next, 2×SYBR Green qPCR Master Mix (#B21203, Bimake, Shanghai, Shanghai, China) was used for RT-qPCR. The primer sequences are listed in **Table 1**. The specific amplification of certain PCR reactions was evaluated using the melting curves. To avoid potential contamination, a negative control reaction was performed in which water was replaced with the cDNA template. Sampling was repeated three times for each well, and the comparative Ct (2−ΔΔCt) value method was used for relative quantification. GAPDH (NM_002046.6) was used as a reference gene.

**Table 1:** Primer used for SYBR Green I qRT-PCR validation.

Gene Symbol	Primer Sequence 5’-3’ (Forward)	Primer Sequence 5’-3’ (Reverse)
**homo-GAPDH**	GGGAAGCTTGTCATCATCAATGG	CATCGCCCCACTTGATTTTG
**homo-IL-1β**	GTACCTGAGCTCGCCAGTG	TGTTTAGGGCCATCAGCTT
**homo-IL-6**	GACAGCCACTCACCTCTTCA	TTCACCAGGCAAGTCTCCTC
**homo-TNF-α**	CCGAGTCTGGGCAGGTCTA	CGTTTGGGAAGGTTGGATG
**homo-ZO-1**	CGGGACTGTTGGTATTGGCTAGA	GGCCAGGGCCATAGTAAAGTTTG
**homo-Claudin-1**	GCGCGATATTTCTTCTTGCAGG	TTCGTACCTGGCATTGACTGG
**homo-Muc-2**	AACGGCCTGCAGAGCTATTC	ATCTTCTGCATGTTCCCAAACTC
**homo-NRF2**	CGACGGAAAGAGTATGAG	TGGGAGTAGTTGGCAGAT
**homo-SOD1**	GACAGCCACTCACCTCTTCA	CTTCATTTCCACCTTTGC
**homo-GPx1**	AAGGTACTACTTATCGAGA ATGTG	GTCAGGCTCGATGTCAATGGTCTG
**homo-NOX2 homo-Keap1 **	AATCCCTGCTCCCACTAACAATGCAGCCAGATCCA	TTTCAAGATGCGTGGAAACTACGCAGAACTGTACCTGTTGA
**homo-Keap1**	ATGCAGCCAGATCCA	GCAGAACTGTACCTGTTGA
**mus-GAPDH**	ACCCCAGCAAGGACACTGAGCAAG	GGCCCCTCCTGTTATTATGGGGGT
**mus-SOD1**	TTCGAGCAGAAGGCAAGCGGTGAAA	AATCCCAATCACACCACAAGCCAA
**mus-SOD2**	CAGACCTGCCTTACGACTATGG	CTCGGTGGCGTTGAGATTGTT
**mus-IL-6**	AGACAAAGCCAGAGTCCTTCAGAGA	GCCACTCCTTCTGTGACTCCAGC
**mus-TNF-α**	AGCAGGCCATCACCACCAAGA	GTGCGTCACATCCTTGAAGTCAT
**mus-NOX2**	TGTTTTCATTTCCTCATCAGAAG	CCAACCACACCAGAATGACA
**mus-Nrf2**	CGAGATATACGCAGGAGAGGTAAGA	GCTCGACAATGTTCTCCAGCTT
**Homo means Homo sapiens and mus means Mus musculus**		

### Establishment of a Mouse LPS Injury Model

Thirty 6-week-old SPF-KM female mice (purchased from the Experimental Animal Center of Huazhong Agricultural University) were randomly divided into five groups: control group, LPS group, KMOS+LPS treatment group, BS+LPS treatment group, and KMOS+BS+LPS treatment group, with 6 mice in each group. All mice were kept in a normal environment with free access to food and water. KMOS was diluted with sterile water to 2 g/L, the gavage group was fed with BS at room temperature, and after washing twice with sterile PBS, the BS and BS + KMOS pre-feeding groups were treated with PBS and resuspended in 2 g/L KMOS and adjusted to a concentration of 1×10^9^ CFU/mL. The control group was gavaged with 200 μL sterile PBS by gavage needle, and the gavage treatment groups were gavaged with 200 μl bacterial solution and konjac mannan oligosaccharide, and the gavage was stopped at 9:00 a.m. every day for 7 days. 200 μl LPS (200 μg/each) was injected intraperitoneally to induce inflammation in mice, and samples were collected 24 hours later.

### Tissue Collection

After 24 h of treatment, the blood was collected by enucleating and blood sampling, and then placed in a refrigerator at 4°C for 2 h. After centrifugation at 3000 g and 4°C for 10 min, the upper clear serum was carefully removed and quickly frozen at -80°C. After blood collection, the mice were euthanized by dislocating the cervical vertebrae, the liver was separated, the surface bloodstains were washed with normal saline pre-cooled to 4°C, and the filter paper was wiped dry. The other part was added with a double volume of pre-cooled normal saline, and the cut tissue was poured into a glass homogenization tube. Next, it was turned up and down dozens of times to fully grind and homogenize the tissue. The prepared homogenate was centrifuged with a low-temperature centrifuge and the supernatant was collected for testing.

Five mice in each group were sacrificed by necking, subjected to aseptic dissection, and then 2 cm of the jejunum, ileum, and colon tissues of the two groups of mice were collected; a part was collected in a cryotube and transferred into liquid nitrogen quickly and stored at -80°C while the remaining 2 cm of the jejunum, ileum, and colon tissues were washed with sterile PBS, cleaned, and soaked in 4% paraformaldehyde for fixation.

### Determination of Antioxidant Enzyme Content

Glutathione peroxidase (GSH-Px) activity can be expressed by the rate of the enzymatic reaction. The enzyme activity can be determined by measuring the consumption of glutathione (GSH) in this enzymatic reaction. GSH reacts with dithiodinitrobenzoic acid to produce the 5-thiodinitrobenzoic acid anion, which presents a relatively stable yellow color. The amount of GSH was calculated by measuring the absorbance at 412 nm. O_2_^-^, produced by the reaction of xanthine and xanthine oxidase, can oxidize hydroxylamine to form nitrite, which appears purple red under the action of a color developer. When the sample contains SOD, it has a specific inhibitory effect on O_2_^-^, reducing the formation of nitrite, and the absorbance decreases when the color is compared at 550 nm. The SOD in the sample was calculated using the vitality formula. The concentration of malondialdehyde in the plasma was determined using thiobarbituric acid colorimetry. Malondialdehyde is the final product of lipid peroxidation in the body. Malondialdehyde can react with thiobarbituric acid to form a red product. The substance has the strongest absorption at 532 nm, so the absorbance value of the reactant can be measured with an ultraviolet-visible spectrophotometer, and the concentration in the measured sample was calculated according to the formula in the manual. The determination of GSH, SOD, T-AOC, and MDA content was performed in accordance with the kit instructions. The kits were purchased from the Nanjing Jiancheng Institute of Biological Engineering.

### H&E Staining of Ileum Tissue

After being euthanized using the neck-inducing method, 2 cm of the ileum tissue was removed and washed with sterile PBS. After cleaning, the samples were immersed in 4% paraformaldehyde for fixation. Then, it was embedded in paraffin, and the sections were stained with alcohol-eosin staining solution.

### Data Statistics

The statistical analysis software GraphPad Prism was used to calculate the means and standard errors of each group of data. Data of each group were expressed as means ± SEM, and t test was used to analyze the significance of the mean difference of relevant groups of data. P<0.05 was marked as significant difference and marked as *. P<0.01 was marked as **; P<0.001 is a very significant difference, marked as ***.

## Results

### Co-Treatment With BS and KMOS Repairs Caco-2 Cell Viability Damage

To study the repair effects of probiotics and KMOS on Caco-2 cell injury, we constructed an LPS-induced intestinal epithelial cell injury model. Caco-2 cells were treated with different concentrations of LPS, and the expression of the related inflammatory factors IL-1β and TNF-α was detected using qPCR. We found that after treatment with 1 μg/mL LPS for 6 h, the expression of the two inflammatory factors was significantly upregulated (**Supplementary Figures S1A, B**), indicating that the cells had an obvious inflammatory response. We also used different concentrations of BS to treat damaged cells and found that the optimal concentration of BS that could repair the damaged cells was 10^7^ CFU/mL. In addition, different concentrations of KMOS promoted the growth of BS, and the growth effect was the best when using KMOS at a concentration of 2 g/L to treat BS for 24 h (**Supplementary Figures S1G–I**).

**Figure 1:**
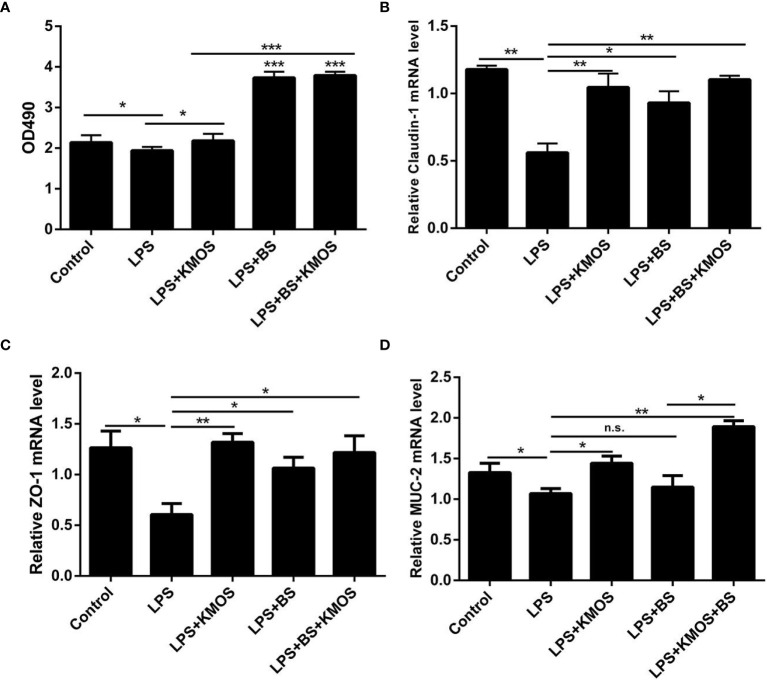
The repair effect of KMOS and BS on cell damage. **(A)** KMOS and probiotics can repair cell activity damage. **(B)** Claudin-1 mRNA expression level. **(C)** ZO-1 mRNA expression level. **(D)** MUC-2 mRNA expression Level. These experiments were repeated three times.*p < 0.05, **p < 0.01 and ***p < 0.001; ns, not significant.

We used the best ratio of KMOS and BS in a culture mixture to achieve the best time of action, and then treated the damaged cells with the mixture. The MTT method was used to detect changes in the activity of Caco-2 cells. The results showed that compared with the LPS treatment group, the Caco-2 cell activity of the KMOS and probiotics co-treated group was significantly upregulated (**Figure 1A**), indicating that KMOS and probiotics worked together to repair the damaged Caco-2 cell activity, and is more conducive for the repair of intestinal epithelial cell activity damage than the konjac mannan oligosaccharide alone, and the effect was significant.

### Co-Treatment With BS and KMOS Repairs Caco-2 Cell Tight Junction Damage

LPS treatment can also cause damage to the tight junctions of Caco-2 cells (28) (**Supplementary Figures S1C–F**), and probiotics can prevent damage to the tight junctions of cells (29, 30). Therefore, we wanted to know whether BS and KMOS have a synergistic repair effect on tight junction damage in Caco-2 cells. We used qPCR to detect changes in the expression of tight junctions ZO-1 and Claudin-1. The results showed that compared with the LPS treatment group, the expression of tight junction ZO-1 and Claudin-1 mRNA in the konjac mannan oligosaccharide and the probiotic co-treatment group was upregulated, and the expression of the tight junction was upregulated compared with the probiotic alone group (**Figures 1B, C**). This shows that the joint action of KMOS and probiotics can repair the damage of the tight junctions of Caco-2 cells, and it is more conducive for the repair of intestinal epithelial cell tight junctions than probiotics alone.

### Co-Treatment With BS and KMOS Repairs the Mucus Barrier Damage of Caco-2 Cells

After treating the cells with LPS, mucin expression in Caco-2 cells decreased, and the cell mucus layer was damaged. We used qPCR to detect MUC-2 mRNA expression. The results showed that compared with the LPS treatment group, the expression of MUC-2 mRNA in the co-treatment group of KMOS and probiotics was significantly upregulated, and the effect was better than that of KMOS and probiotics alone (**Figure 1D**).

### Co-Treatment With BS and KMOS Repairs Oxidative Damage in Caco-2 Cells

LPS can also cause oxidative damage in cells (10). To test whether BS and KMOS can repair cell oxidative damage, we constructed a Caco-2 cell oxidative damage. We used different concentrations of LPS to treat Caco-2 cells to detect the expression level of GSH and found that when the stimulating concentration of LPS was 2μg/mL and the action time was 8 h, the GSH expression was the lowest (**Supplementary Figure S2A**), and NOX2 expression was significantly upregulated (**Supplementary Figure S2B**), SOD enzyme activity was significantly reduced (**Supplementary Figure S2C**), and MDA oxidation level increased significantly (**Supplementary Figure S2D**). Therefore, the above-mentioned concentration and treatment times are oxidative damage conditions for cells.

**Figure 2:**
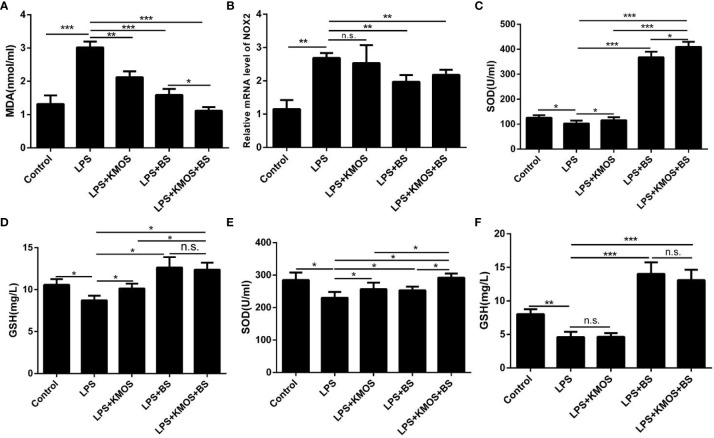
Co-treatment with BS and KMOS on the repair of oxidative damage in Caco-2 cells. The level of cell **(A)** MDA in different treatment groups. **(B)** The expression level of NOX2. **(C)** SOD enzyme activity in cell culture supernatant. **(D)** The expression level of GSH. **(E)** SOD enzyme activity in cell lysate. **(F)** GSH content. These experiments were repeated three times. *p < 0.05, **p < 0.01 and ***p < 0.001; ns, not significant.

Then, we used the optimal ratio of KMOS and BS to mix the culture and then treated the damaged cells after reaching the optimal growth concentration. The results showed that the addition of BS and oligosaccharide mixtures significantly increased compared to the LPS treatment group. Down-regulation of the level of oxidation marker MDA (**Figure 2A**) and the expression of NADPH oxidase subtype NOX2 (**Figure 2B**), and the combination of the two has a better effect on cell repair. We also tested the SOD enzyme activity and GSH content in the cell culture supernatant and cell lysate and found that the SOD enzyme activity in the cell culture supernatant of the KMOS and probiotics co-treatment group was significantly upregulated (**Figure 2C**), and the GSH content was significantly increased (**Figure 2D**), and the SOD enzyme activity and GSH content in the cell lysate were similar to those in the supernatant (**Figures 2E, F**).

**Figure 3:**
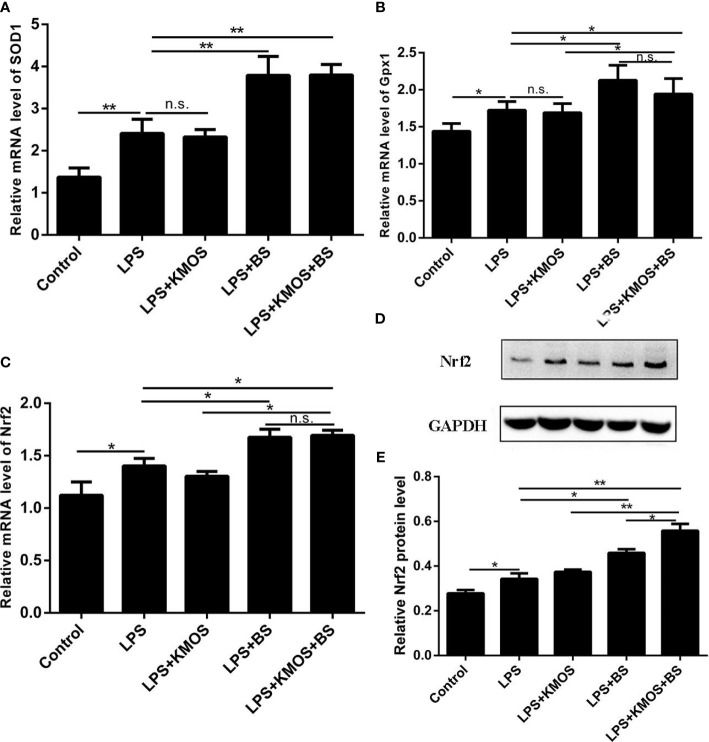
The co-treatment of BS and KMOS can repair the oxidative damage of Caco-2 cells. Cell **(A)** SOD1 mRNA expression level in different treatment groups. **(B)** Gpx1 mRNA expression level. **(C)** The expression level of Nrf2 mRNA. **(D)** Western blot result of Nrf2 **(E)** Level of Nrf2 protein. These experiments were repeated three times. *p < 0.05 and **p < 0.01; ns, not significant.

Next, we tested the expression levels of SOD1, Gpx1, and Nrf2 at the mRNA level, and found that the expression of SOD, Gpx1, and Nrf2 in the combined treatment group of probiotics and oligosaccharides was upregulated compared with the LPS-injured group (**Figures 3A–C**), but compared with the probiotics alone, there was no significant difference in the addition group. We also used western blotting to detect the expression of Nrf2 protein and found that the combined treatment of oligosaccharides and probiotics can significantly increase the expression of Nrf2 protein compared to the LPS alone injury group (**Figures 3D, E**).

### Protective Effects of KMOS and BS on LPS-Induced Liver Injury in Mice

**Figure 4:**
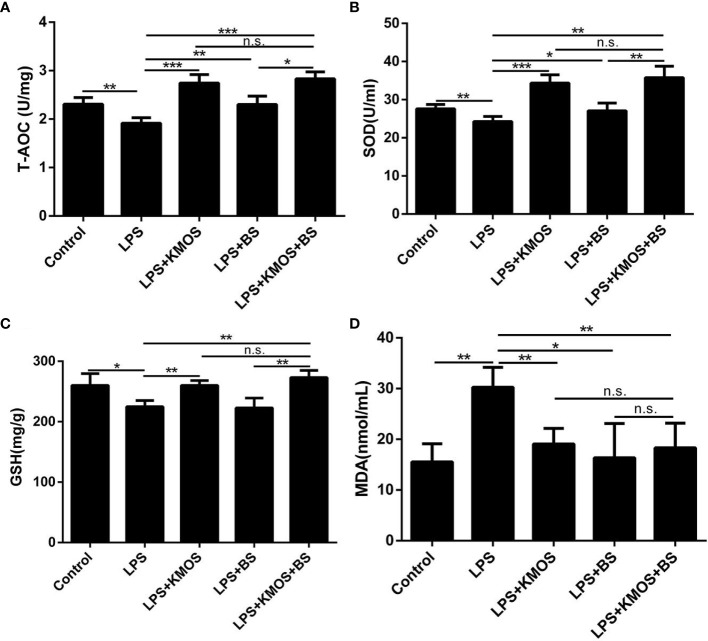
The protective effects of KMOS and BS on LPS-induced liver injury in mice. Liver tissue **(A)** T-AOC enzyme activity of mice in different treatment groups. **(B)** SOD enzyme activity. **(C)** GSH enzyme activity. **(D)** MDA content. These experiments were repeated three times. *p < 0.05, **p < 0.01 and ***p < 0.001; ns, not significant.

The above studies indicate that KMOS and BS have a synergistic repair effect on cell damage, but it is still unknown whether this synergistic repair functions *in vivo*. To study its synergistic repair effects *in vivo*, we administered BS, KMOS, and their mixtures to the stomach for 7 consecutive days, and then injected LPS into the intraperitoneal cavity to establish a mouse injury model. The mice were sacrificed 24 h later. In mice, the liver was collected to determine relevant antioxidant enzyme indices. The results showed that compared with the LPS treatment group, the pre-infused gastric oligosaccharide group significantly increased the activities of the three antioxidant enzymes (**Figures 4A–C**). The group of gastric probiotics only significantly improved the activities of superoxide dismutase and total antioxidant enzymes, but had no significant effect on the content of GSH in the liver (**Figures 4A–C**). The combined treatment with KMOS and BS significantly improved the three antioxidant enzymes in the liver, and the effect was more significant than that of the group treated with probiotics alone (**Figures 4A–C**). We also tested the MDA levels in the serum of mice. The results showed that the serum MDA of mice in the LPS alone treatment group increased significantly, and both KMOS and BS gavage could significantly reduce the MDA content in the serum of LPS-treated mice. The effect of the combined gavage was more significant (**Figure 4D**).

### Protective Effects of KMOS and BS on LPS-Induced Ileal Tissue Damage in Mice

**Figure 5:**
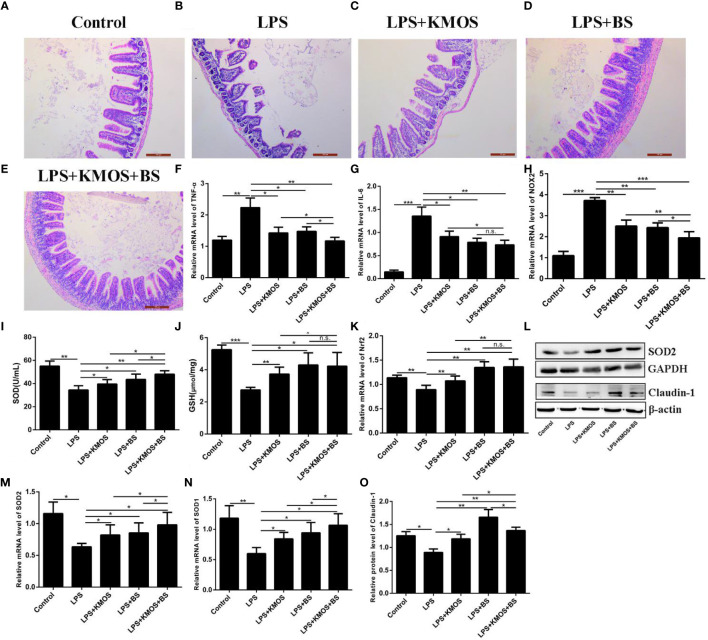
The protective effects of KMOS and BS on LPS-induced ileal tissue damage in mice. Mouse ileum tissue **(A–E)** H&E staining in different treatment groups, **(F)** TNF-α mRNA expression level, **(G)** IL-6 mRNA expression level, **(H)** NOX2 mRNA expression level, **(I)** SOD enzyme activity, **(J)** GSH enzyme activity, **(K)** Nrf2 mRNA expression Level, **(L)** Western blot result of SOD2 and Claudin-1, **(M)** SOD2 mRNA expression Level, **(N)** SOD1 mRNA expression level, **(O)** Claudin-1 protein level. *p < 0.05, **p < 0.01 and ***p < 0.001; ns, not significant.

Studies have shown that LPS treatment can damage the mouse ileum tissue (31). Therefore, we wanted to know whether KMOS and BS are beneficial for repairing damaged ileum tissue in mice. We performed H&E staining on the ileum tissue of each group of mice (**Figures 5A–E**). We found that LPS caused obvious damage to the ileum tissue of the mouse, with broken intestinal villi and tissue pyknosis (**Figure 5B**). The addition of KMOS and BS protected the mouse ileum tissue from the damage to varying degrees (**Figures 5C–E**).

To study the protective effects of oligosaccharides and probiotics on mouse intestinal inflammatory damage, we collected mouse ileum tissues and used RT-qPCR to detect changes in the expression of inflammation-related genes in mouse ileum tissues. The results showed that compared with the LPS treatment group, the expression of TNF-α was downregulated in the oligosaccharide and probiotic gavage groups, and the combined gavage group was more downregulated (**Figure 5F**). The IL-6 gene was significantly upregulated after LPS treatment. Pretreatment with BS and KMOS significantly inhibited LPS-induced upregulation of inflammatory factors in mouse ileum tissue caused by LPS (**Figure 5G**), indicating that oligosaccharides and probiotics are effective in mice, and inflammatory damage in the intestine has a synergistic repair effect.

Next, we studied the protective effects of oligosaccharides and probiotics on oxidative damage in the mouse intestine. We tested the effects of oligosaccharide and probiotic mouse gavage on mouse intestinal oxidase genes. The data showed that the expression of the oxidation gene NOX2 in the intestinal cells of mice in the LPS alone treatment group was significantly upregulated (**Figure 5H**), and the groups of gavage KMOS and BS could significantly inhibit the upregulation of the oxidation gene NOX2 caused by LPS And the difference in downregulation of the two combined gavage groups was more significant than that of single gavage (**Figure 5H**). We then tested the changes in antioxidant enzymes and genes in the mouse intestines and found that the SOD enzyme activity and GSH content of mice in the LPS alone treatment group were significantly downregulated, and the groups of BS and KMOS were administered with the two antioxidants. Oxidase activity was significantly upregulated (**Figures 5I, J**). RT-qPCR was used to detect the expression of Nrf2, SOD1, and SOD2. The results showed that, compared with the blank control group, the acute oxidative damage induced by a high concentration of LPS destroyed the antioxidant enzyme system in the mouse intestine and inhibited the expression of mouse Nrf2, SOD1, and SOD2 genes (**Figures 5K, M, N**). Compared with the LPS treatment group, BS and Konjac mannan oligosaccharide pretreatment significantly increased the expression of the antioxidant genes. Western blot analysis revealed that the expression of SOD2 protein was consistent with the transcription level (**Figure 5L**). This shows that both BS and KMOS can significantly upregulate the expression of antioxidant genes in the mouse intestine, improve the antioxidant capacity of mice, and have a good protective effect against oxidative damage in mice.

Finally, we studied the protective effects of oligosaccharides and probiotics on LPS-induced intestinal tight junction protein damage in mice. Our data showed that the expression of Claudin-1 protein was significantly downregulated in the LPS treatment group, while the expression of Claudin-1 protein in the group supplemented with BS and KMOS was significantly upregulated (**Figures 5L, O**), indicating that it has a good protective effect on the intestinal tight junction damage caused by LPS.

## Discussion

The intestine is the largest reservoir of bacteria and endotoxins in the human body. Intestinal epithelial cells provide a physical barrier for the body, which can protect the body from microbial invasion (32). Previous studies have shown that KMOS is effective in keeping the intestinal mucosa tight and intact, improving immunity (8), regulating the balance of intestinal flora, and improving intestinal function (33). By interacting with microorganisms, BS can promote intestinal barrier function and maintain the integrity of the intestinal epithelial barrier (34). However, whether KMOS and BS have a synergistic repair effect on intestinal injury remains unknown. In this study, we used the LPS-induced Caco-2 cell and mouse intestinal injury model to study the synergistic effects of KMOS and BS on Caco-2 cells and intestinal injury. Our research results show that the combined use of KMOS and BS has a synergistic repair effect on Caco-2 cells and mouse intestinal injury, and can enhance the antioxidant function of cells, which is beneficial for LPS-induced acute injury in mouse models.

LPS is a biologically active bacterial structural component released after the death and disintegration of bacteria and is the main pathogenic factor of bacteria (35). LPS acts on intestinal epithelial cells and can increase the permeability of intestinal epithelial cells (35, 36), increases the expression of related inflammatory cytokines, causes inflammation (36–38), and inhibits the expression of tight junction proteins in intestinal epithelial cells (38), resulting in damage to the intestinal barrier. Intestinal homeostasis is precisely regulated by a variety of cytokines (39). The tightly linked proteins ZO-1 and ZO-2 can bind directly to cytoskeletal proteins, thereby regulating cellular tissue and epithelial morphogenesis (7, 40). Deletion of ZO-1 can increase intestinal epithelial permeability and promote the development of intestinal inflammation (41, 42). It has been reported that the probiotic B. subtilis CW14 repairs the epithelial barrier and reduces the toxicity of ochratoxin A to Caco-2 cells by promoting the expression of ZO-1 protein (27). Claudin-1 is also an important tight junction protein and helps regulate intestinal epithelial homeostasis by regulating Notch signaling (43). Intake of BS in mice upregulated the expression of TJ proteins (claudin-1, and ZO-1), thereby repairing intestinal barrier function (44), which is consistent with our findings. Mucins are major components of mucus and form a protective barrier between the resident microbiota and the underlying immune cells in the intestine. Oral administration of Bacillus subtilis fermented milk promotes the expression of MUC-2 in inflammatory epithelial cells and may play a role in the treatment of dextran sulfate sodium salt (DSS)-induced IBD (27). This repair effect may be due to some bioactive peptides produced by bovine β-casein of Bacillus subtilis acting through the protective function of Mucin2 (27). The enhanced expression of MUC-2 by combined treatment of KMOS and BS to repair LPS-induced adhesive damage may be due to the role of bioactive substances in this repair process. Metabolites of BS, such as surfactant A and polyγ-glutamic acid, have been reported to alleviate symptoms in animal models of IBD (45). In addition, nattokinase of BS inhibits inflammation and oxidative stress in mice (46).

Bacillus subtilis fermented milk inhibits the expression of the pro-inflammatory cytokine TNF and promotes the expression of anti-inflammatory cytokines, thereby reducing local inflammatory damage to the intestinal mucosa (25). Overexpression of TNF leads to apoptosis of intestinal epithelial cells (IECs). In contrast, the anti-inflammatory factor IL-6 is usually associated with inflammation and promotes proliferation and repair of the intestinal epithelium (39). The expression of TNF-α in epithelial cells could be inhibited by the synergistic effect of KMOS and BS, which might inhibit apoptosis of intestinal epithelial cells and thus alleviate the injury. In contrast, the expression of IL-6 was lower in the KMOS and BS co-treatment group, indicating the inhibition of intestinal inflammation (16).

Intestinal cells sense LPS and other pathogenic receptors of intestinal flora through pattern recognition receptors, promote the expression of NADPH oxidase and the activity of reactive oxygen species, and produce a large number of reactive oxygen species (47, 48). Although the generated active oxygen can kill pathogenic bacteria, the attack of a large amount of active oxygen on the intestinal epithelium also causes oxidative damage to the intestinal epithelium (49) and causes damage to the structure and function of the intestine (50–52). The precondition for cells to exert their antioxidative stress effect is to activate Nrf2. When the body is oxidatively damaged, the expression of Nrf2 increases, and the expression of downstream antioxidants such as SOD1, HO-1, and GPX1 is upregulated to combat free radical damage. In addition, the KMOS treatment group had no significant effect on antioxidant factors at the transcriptional level. We speculate that the antioxidant action mechanism of KMOS may be clearing active oxygen free radicals through its own physical and chemical properties because some plant polysaccharides, such as APS and garlic polysaccharides, scavenge free radicals and improve the activity of antioxidant enzymes (39, 40). The antioxidant system of the human body is inextricably linked to the immune system. Oxidative stress is often accompanied by inflammation (53). Overexpression of TNF leads to apoptosis of intestinable epithelial cells (IECs) (39). Bacillus subtilis fermented milk can inhibit the expression of pro-inflammatory cytokines TNF and promote the expression of anti-inflammatory cytokines, thus reducing local inflammatory damage in the intestinal mucosa (25). The expression of TNF-α in epithelial cells can be inhibited by the synergistic effect of KMOS and BS, which may inhibit apoptosis of intestinal epithelial cells thereby alleviating the injury. The anti-inflammatory factor IL-6 is usually associated with inflammation and may promote proliferation and repair of intestinal epithelium (39). In contrast, the expression of IL-6 was lower in the KMOS and BS co-treatment groups, indicating the inhibition of intestinal inflammation (25).

Inflammatory damage in the intestine has been reported to be associated with a decrease in the abundance and diversity of the intestinal microbiota (54). Whether oral administration of KMOS and BS altered the composition of the intestinal flora in mice in this study remains to be further investigated, and we hypothesize that KMOS and BS administration may alter the intestinal flora diversity in mice. Because it has been shown in other studies that oral administration of Bacillus subtilis fermented milk can significantly increase the variety and diversity of intestinal microbiota (25). As for the mechanism of how KMOS affects intestinal microbial diversity, more in-depth studies are needed. In animal experiments, KMOS could affect the antioxidant enzyme system in the intestine of mice, and there was a significant effect of oxidative enzymes, which may be related to the utilization of KMOS by microorganisms in the intestine (55). In conclusion, our study suggests that the combined use of KMOS and BS can help improve LPS-induced intestinal damage in mice and is expected to be a potential novel combination functional food.

## Data Availability Statement

The original contributions presented in the study are included in the article/**Supplementary Material**, further inquiries can be directed to the corresponding author/s.

## Ethics Statement

The animal study was reviewed and approved by the Institutional Animal Care and Use Committee of Huazhong Agricultural University.

## Author Contributions

JW conceived and designed the research. SZ and SW conducted the experiments. LC analyzed data. LC and JW wrote the manuscript. All authors contributed to the article and approved the submitted version.

## Funding

This work was supported by the Fundamental Research Funds for the Central Universities (Program No. 2662019FW012), the National Key R&D Program of China (Program No. 2018YFD0500204) and the Science and Technology Program of Wuhan, China (Program No. 2016020101010091).

## Conflict of Interest

The authors declare that the research was conducted in the absence of any commercial or financial relationships that could be construed as a potential conflict of interest.

## Publisher’s Note

All claims expressed in this article are solely those of the authors and do not necessarily represent those of their affiliated organizations, or those of the publisher, the editors and the reviewers. Any product that may be evaluated in this article, or claim that may be made by its manufacturer, is not guaranteed or endorsed by the publisher.
