# Supplementary material for: Synergistic Protective Effect of Konjac Mannan Oligosaccharides and Bacillus subtilis on Intestinal Epithelial Barrier Dysfunction in Caco-2 Cell Model and Mice Model of Lipopolysaccharide Stimulation
Source: Front Immunol. 2021 Sep 17;12:696148. doi: 10.3389/fimmu.2021.696148 (PMC8484872; doi:10.3389/fimmu.2021.696148)
Supplement: Supplementary file 1 [file DataSheet_1.docx]

Supplementary Material

## Supplementary Figures


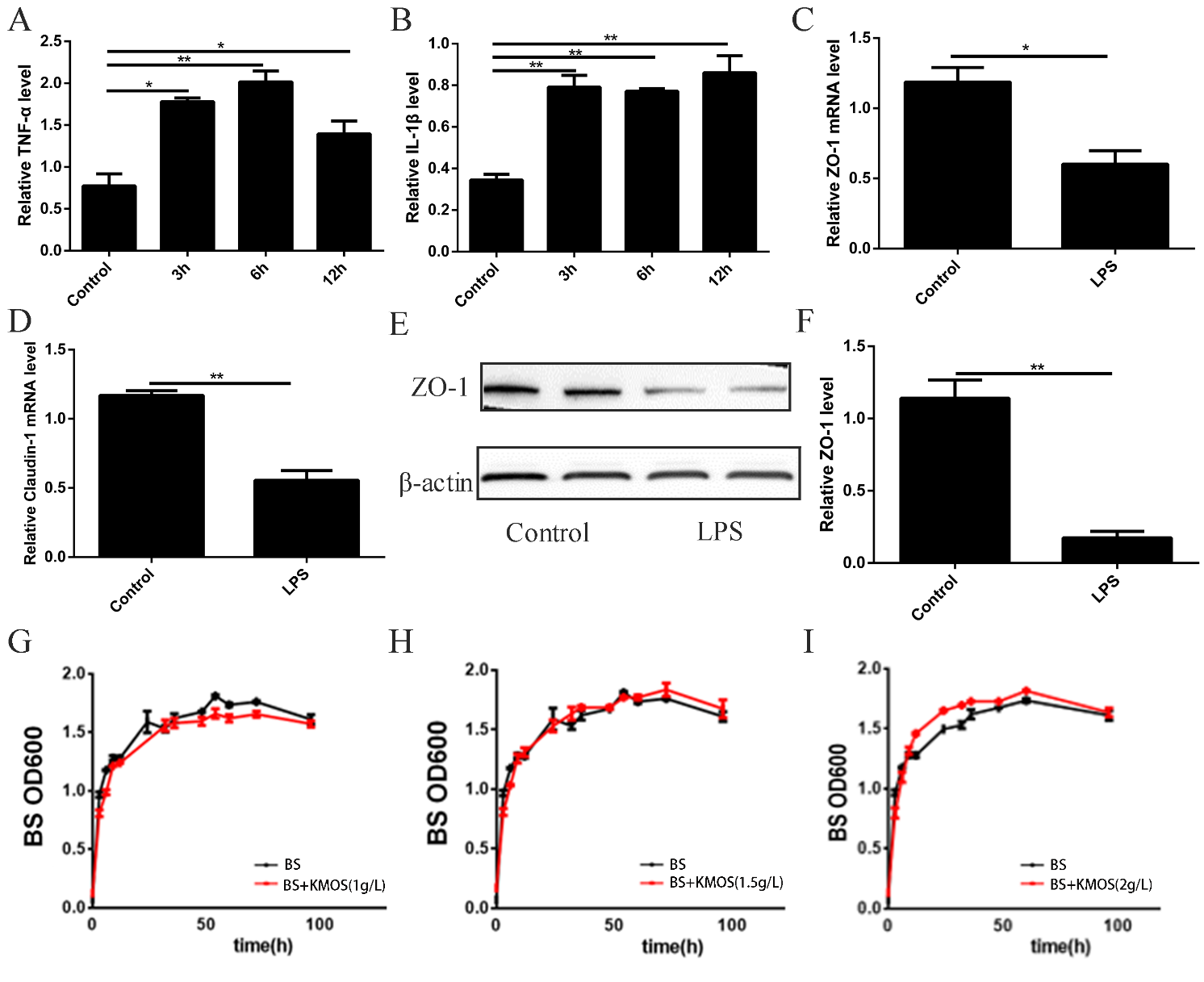


**Supplementary Figure 1.** Figure S1. Cell injury model construction. (A) TNF-α expression level. (B) IL-1β expression level. (C) ZO-1mRNA expression level. (D) Claudin-1 mRNA expression level. (E) ZO-1 protein expression level. (F) ZO-1 protein expression level. (G-I) BS growth curve treated with different concentrations of KMOS. These experiments were repeated three times. *p<0.05; and **p<0.01.


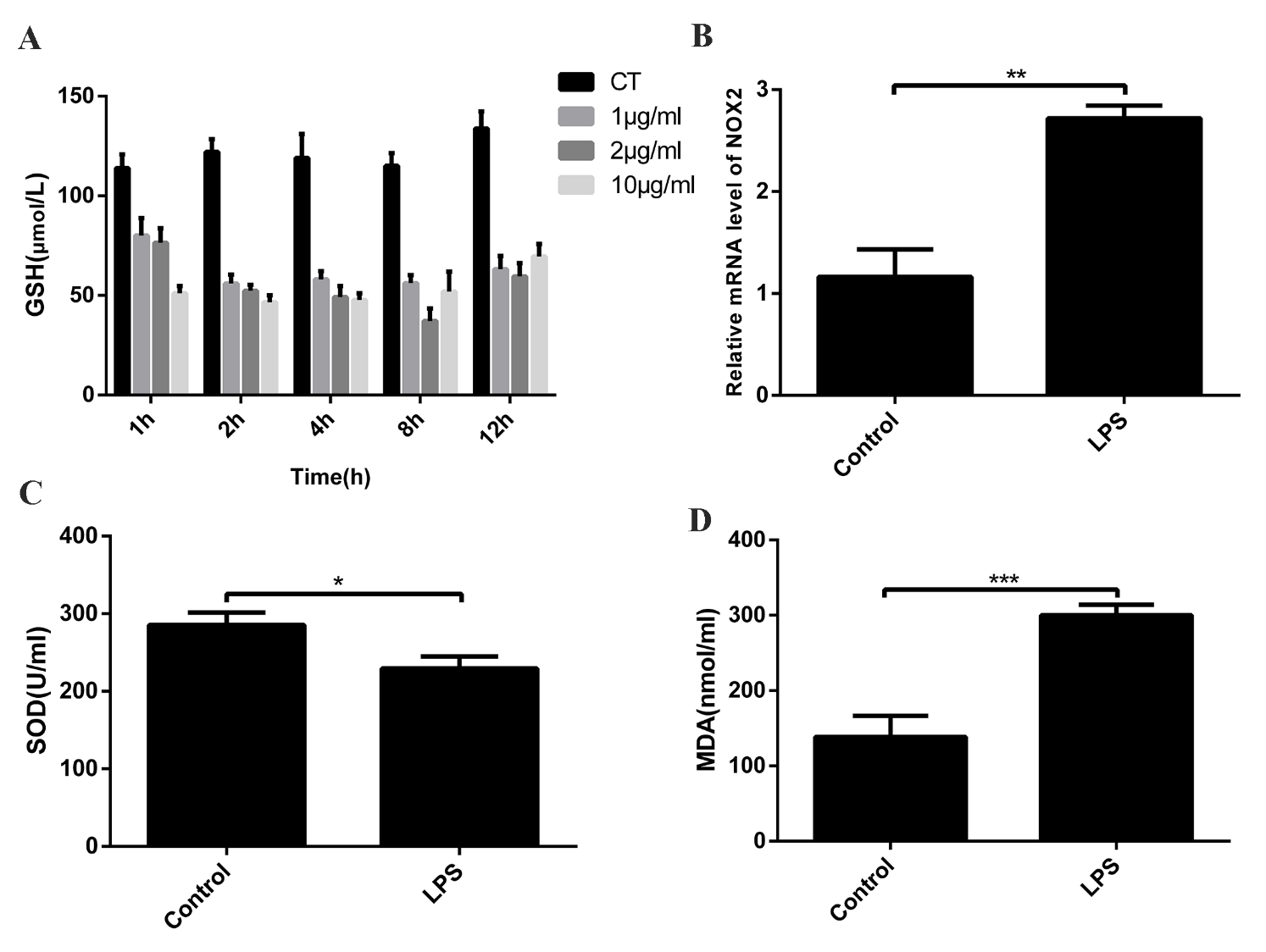


**Supplementary Figure 2**. LPS treatment causes oxidative damage to cells. (A) The expression level of GSH in cells treated with different concentrations of LPS over time. (B) The expression of NOX2 in cells after LPS treatment. (C) Cell SOD activity after LPS treatment. (D) The level of cell oxidation marker MDA after LPS treatment. These experiments were repeated three times. *p<0.05; and **p<0.01.
